# Epigenetic Mechanisms Associated with Livestock Adaptation to Heat Stress

**DOI:** 10.3390/biology14091154

**Published:** 2025-09-01

**Authors:** Sundar Aravindh, Mullakkalparambil Velayudhan Silpa, Santhi Priya Voggu, Ebenezer Binuni Rebez, Gajendirane Kalaignazhal, Mouttou Vivek Srinivas, Frank Rowland Dunshea, Veerasamy Sejian

**Affiliations:** 1 College of Veterinary Science and Animal Husbandry, Odisha University of Agriculture and Technology, Bhubaneshwar 751003, India; aravindh.sundar2017@gmail.com (S.A.); gnazhal99@gmail.com (G.K.); 2 Rajiv Gandhi Institute of Veterinary Education and Research, Puducherry 605009, India; binunirebez.e@gmail.com (E.B.R.); vivekvet24@gmail.com (M.V.S.); 3 Department of Animal Science, University of Connecticut, Storrs, CT 06269-4040, USA; santhi_priya.voggu@uconn.edu; 4 School of Agriculture, Food and Ecosystem Sciences, Faculty of Science, The University of Melbourne, Parkville, Melbourne, VIC 3010, Australia; fdunshea@unimelb.edu.au; 5 Centre for Climate Resilient Animal Adaptation Studies, ICAR-National Institute of Animal Nutrition and Physiology, Bangalore 560030, India; drsejian@gmail.com

**Keywords:** adaptation, climate change, DNA methylation, epigenetics, gene expression

## Abstract

The livestock sector, a crucial source of revenue and global food security, is facing serious challenges due to climate change driven by global warming. This leads to serious effects on animal health and productivity, making it difficult for the livestock industry to meet the global demand and sustain the livelihoods of farmers. The main factor affecting livestock’s productivity is heat stress. However, animals develop various adaptive mechanisms to cope with the effects of heat stress. Cellular and molecular responses act as key defense mechanisms, enabling animals adapt to environmental changes. The recent advancements in molecular biology have opened up opportunities for extensive research on epigenetics, which has a key role in regulating gene expression in animals in response to environmental stimuli. Such studies have gained considerable attention regarding heat acclimation in animals due to the fact that epigenetic mechanisms have been recognized as key players in long-term adaptation to high temperatures in farm animals. This review summarizes the different mechanisms and methodologies used to assess heat stress-associated epigenetic changes, including DNA methylation, which is an extensively studied epigenetic regulatory mechanism in relation to gene expression. The review also highlights the mechanisms and regulation of adaptation to heat stress in animals and collates information related to various epigenetic markers to assess the heat stress response, thereby aiding in improving thermal resilience in animals.

## Simple Summary

Heat stress, a major consequence of global climate change, has a deleterious impact on livestock production, often manifested as declined productivity, impaired welfare, fertility, susceptibility to disease and mortality. Animals exhibit several responses to heat stress, which aid in assessing their relative susceptibility and/or resilience to heat stress. There is a scarcity of information on the underlying mechanisms of several such responses, and improved comprehension of its hidden intricacies, especially of cellular and molecular changes, is critical in developing effective mitigation strategies. Epigenetic modifications are among the major gene-regulatory mechanisms that link environmental factors and animal responses. Such genomic modifications do not change the nucleotide sequence; however, they are stated to have transgenerational effects. This review summarizes the mechanisms and methodologies employed to assess heat stress-associated epigenetic changes in livestock. Based on the compilation of the literature, the review also highlights the various epigenetic markers that can be used to assess the heat stress response in livestock. Research on epigenetic responses to heat stress in livestock is still at its infancy; hence, encouraging the adoption of this approach, in collaboration with other biotechnological methods, may be considered to achieve a holistic assessment of heat tolerance and/or susceptibility in livestock.

## 1. Introduction

The livestock sector is highly valued and has served as a fundamental source of income for the rural stratum and played a crucial role in ensuring global food security. However, the climate change phenomenon has been a long-standing challenge to the livestock sector. The global warming-associated changes in the mean climatic variables have significant impacts on animal health and production, thereby undermining the potential of livestock systems to sustain livelihoods and meet the surging global demand for livestock products [1]. As reported by the Intergovernmental Panel on Climate Change (IPCC), the projected risks and losses from climate change will be elevated with the increment in global warming [2]. This escalation will pose a significant challenge to the abilities of the livestock sector in contributing to the food supply and broader sustainability goals.

Global warming-associated heat stress in livestock and its implications have been underlined in the literature, as they act as major factors hindering livestock production [3,4]. However, animals adapt to thermal challenges through gradual modifications that occur over successive generations [3]. Hence, it is critical to find effective solutions by improving the comprehension of livestock’s adaptability to climate change. Besides morphological, behavioral, physiological, blood–biochemical and endocrine-adaptive responses, cellular and molecular adaptations are crucial in the survival and welfare of animals in challenging environments [5]. Furthermore, it is established that cellular and molecular adaptation pathways are cardinal mechanisms that play a central role in enabling animals to cope with stressors [5]. Thus, the improved comprehension of the hidden intricacies of cellular and molecular changes under heat stress conditions is critical in developing effective mitigation strategies.

In recent years, the epigenetic features that control gene expression in animals have garnered significant attention. The epigenetic machinery has been associated with the heat acclimation process in farm animals, with evidence suggesting that epigenetic changes contribute to long-term adaptation to elevated temperatures [6,7]. As epigenetic modifications are considered major gene-regulatory mechanisms linking environmental factors and animal responses, it is crucial to identify epigenetic mechanisms and quantify epigenetic changes in heat-stressed livestock. This review summarizes the mechanisms and methodologies employed to assess heat stress-associated epigenetic changes. The exposure of animals to heat stress has been suggested to alter gene methylation patterns. In this regard, DNA methylation is currently the most extensively studied epigenetic regulatory mechanism in relation to gene expression. Thus, this article summarizes updated information on various methylome patterns in heat stress-adapted livestock. Furthermore, the article highlights other essential epigenetic mechanisms, such as histone modification, chromatin remodeling and non-coding RNA and its regulation of adaptation to various stressors. The review further attempts to collate information related to various epigenetic markers that can be used to assess the heat stress response in livestock.

## 2. Heat Stress as the Major Factor Influencing Livestock Production

Global agricultural sustainability has been deeply impacted by the long-term effects of climate change in recent decades. As reported by the Food and Agriculture Organization (FAO), climate change poses direct and indirect threats to agri-food systems because of changing temperatures and rainfall patterns, increased frequencies of extreme weather events and ocean acidification [8]. These changes negatively impact livestock performance, and modeling studies generally predict adverse outcomes [9]. Likewise, as the climate crisis intensifies, it has been established to have a marked impact on livestock feeding, production and reproduction, with serious economic consequences for the animal agricultural sector [10].

Furthermore, it has been reported that global warming will continue to intensity (2021–2040), and the temperature increase is likely to reach 1.5 °C under very low greenhouse gas (GHG) emissions and is likely or very likely to exceed 1.5 °C under higher-emissions scenarios [2]. Thus, warming-associated heat stress is widely established as one of the most significant environmental stressors influencing livestock productivity and welfare. The direct impacts of alarming changes in climatic variables and the increased frequency of warming episodes include negative influences on animals’ thermoregulatory systems, metabolism, immune status and reproductive abilities [9].

Heat stress arises as a result of environmental conditions that challenge an animal’s thermoregulatory system and its functioning [4]. High ambient temperatures, humidity and radiant energy impact heat dissipation in livestock, leading to an increased body temperature, which subsequently initiates compensatory mechanisms to reestablish homeostasis [11]. Consequently, heat stress effects are manifested as a reduction in livestock productivity, welfare and fertility and greater susceptibility to disease and mortality [1], causing serious economic repercussions.

Heat stress also causes a decrease in feed intake, milk production, body weight gain, growth rates, egg production, reproductive efficiency, feed conversion efficiency and animal performance [11,12]. Thus, heat stress has a detrimental impact on livestock production and consequently global food security.

## 3. Significance of Cellular and Molecular Changes Associated with Livestock Adaptation

Exposing animals to extreme temperatures initiates a cascade of cellular and molecular responses that interfere with the normal functioning of cells to maintain homeostasis [13]. Heat stress causes a number of cellular and molecular responses, like the production of heat shock proteins (HSPs), the activation of antioxidant enzymes and alterations in gene expression, metabolic pathways like cell survival pathways and other mechanisms, including DNA synthesis, replication and repair, cellular division and the activity of nuclear enzymes and DNA polymerases [3]. These responses serve as vital defense mechanisms that enable animals to adapt to and withstand environmental fluctuations [14]. However, this is a graded reaction, and its characteristics depend upon the extent and duration of cellular damage and heat exposure [15].

Stress-induced HSPs and heat shock factors (HSFs) are the main components of the molecular networks behind the cellular response to heat stress [16]. Heat stress causes the protein structure to be disrupted, leading unfolded proteins to aggregate [3]. However, the cells respond through the production of HSPs, which function as molecular chaperones, helping to stabilize the disrupted proteins, assist in protein folding and facilitate their transport across membranes. These HSPs also influence thermotolerance and antioxidant defenses and activate survival mechanisms to remove damaged proteins, thereby protecting the cells against cellular damage [17].

Additionally, heat stress impairs the stability and fluidity of cellular membranes, as well as the function of transmembrane transport proteins and receptors [18], which will be restored by cellular responses regulating ion channels, osmolyte concentrations and metabolic pathways to maintain homeostasis. Furthermore, there are alterations in the expression levels of several genes associated with heat stress response and adaptation [19]. There are also other changes, like epigenetic modifications, which are heritable and contribute to long-term adaptation to environmental stresses.

Recent advancements in molecular and biotechnological tools have significantly enhanced the understanding of the cellular and molecular mechanisms underlying complex adaptation traits [20]. Thus, a thorough understanding of the mechanisms associated with environmental adaptation using these technologies will help in better elucidating their responses and identifying biomarkers that can be incorporated into breeding programs to identify and develop resilient animals, thereby improving livestock’s productivity and health amid climatic shifts.

## 4. Different Epigenetic Mechanisms in Livestock in Response to Environmental Stressors

**Figure 1:**
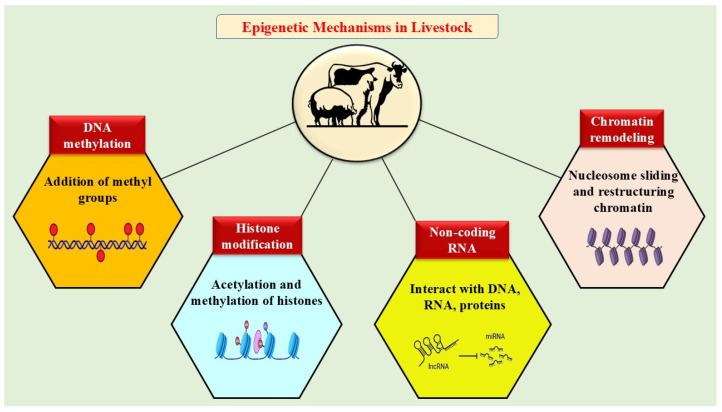
Overview of the different epigenetic mechanisms in livestock.

Epigenetics studies the heritable molecular changes that control gene expression and genome functions, causing variations in the phenotype without modifying the underlying DNA sequence [21]. Thus, it plays an important role in determining phenotypic variation through its complex interactions with the genetic composition, environmental factors and other non-genetic influences [22]. Adaptation is a complex trait that involves various epigenetic modifications that impact the accessibility of DNA to transcription factors, which subsequently control transcriptional activity and alter phenotypic behavior [7]. Various studies have shown that environmental stressors, especially heat stress, alter the epigenetic mechanisms that enable animals to adapt and change their phenotypes in response to environmental stimuli, which could have an adverse impact on the animal’s productivity [6,7]. The different epigenetic mechanisms include DNA methylation, histone modifications, the regulation of non-coding RNA (ncRNA) and chromatin remodeling, which regulates gene expression and is essential for the stability and functionality of the genome [23] (Figure 1).

### 4.1. DNA Methylation

DNA methylation is the earliest-discovered and most important epigenetic mechanism and has been widely studied. It usually occurs at cytosine-phosphate-guanine (CpG) dinucleotides and rarely in non-CpG sites [24,25] and involves the addition of a methyl group to the 5′ carbon position of the pyrimidine cytosine ring by DNA methyltransferases to yield 5-methylcytosine (5^m^C) [26]. Various biological processes, such as gene expression regulation, developmental regulation, X chromosome inactivation, gene imprinting, cell differentiation and aging, depend on DNA methylation [27]. Studies mapping DNA methylome patterns through bisulfite sequencing analysis have aided in better understanding the epigenetic mechanisms that regulate gene expression in response to heat stress, related to production, reproduction, the immune response and adaptation in animals [28]. For example, a comparative study between indicine and crossbred cattle, evaluating their DNA methylation profiles, revealed 4599 significantly differentially methylated CpGs in indicine compared to crossbred cattle [7]. The authors suggested that this epigenetic difference might be the reason for Hariana (indicine) cattle’s high degree of thermotolerance and long-term adaptation to tropical temperatures [7]. Furthermore, a study attributed the reduction in sperm quality during the summer season to differentially methylated regions detected through bisulfite sequencing [29], while another study explained reduced growth rates during heat stress being due to similar epigenetic changes [30].

### 4.2. Histone Modifications

Histones are a group of proteins that bind and organize DNA molecules into compact structural units known as nucleosomes [31]. Common histone modifications include methylation, acetylation, phosphorylation and ubiquitylation, which are either deposited on or removed from histones by particular enzymes [31]. Numerous post-translational modifications (PTMs) that occur in histones have the potential to carry epigenetic information. These PTMs have the ability to change the charge states of histones, which consequently control the remodeling of the chromatin structure, transcription factor access and the recruitment of certain binding proteins. Consequently, environmental variables have the potential to dynamically modify PTMs, resulting in differential gene expression and translation into phenotypic plasticity and acclimatization [32]. In a study conducted in layer chickens, histone modification analysis revealed that a modification in the adrenal H3K27me3 linked to endocrine function could have contributed to the thermotolerance of the chickens [32]. In another study, the authors observed reduced developmental potential in bovine oocytes during heat stress because of decreased gene expression levels due to epigenetic modifications on histone proteins [33].

### 4.3. Chromatin Remodeling

Besides DNA methylation and histone modification, another significant factor influencing the chromatin structure is nucleosome remodeling. Chromatin remodeling complexes regulate nucleosome formation, which allows proper DNA compaction and chromatin activity [23]. It is primarily performed by Adenosine Triphosphate (ATP)-dependent complexes and involves the rearranging or reorganizing of nucleosomes inside chromatin to either promote or prevent access to the surrounding DNA. This contributes to epigenetic regulatory function in a number of important biological processes, such as apoptosis, development, pluripotency and the replication and repair of DNA [34]. There are studies highlighting the changes in chromatin remodeling during heat stress, which alter gene expression. A study in heat-stressed spermatozoa from bulls highlighted that heat stress altered chromatin condensation in sperm cells. This negatively impacted the DNA methylation pattern in sperm cells, which is necessary for normal zygote development. Thus, the authors concluded that alterations in chromatin condensation could be the reason for the decrease in the fertilization potential of heat-stressed spermatozoa [35].

### 4.4. Non-Coding RNAs

Another important epigenetic mechanism impacting livestock production and health by regulating gene expression at the transcriptional and post-transcriptional levels is non-coding RNAs [23]. Despite not coding for proteins, these non-coding RNA molecules have the ability to influence gene expression through interacting with DNA, RNA and proteins, changing critical mechanisms associated with important economic traits [23]. ncRNAs can be broadly divided into long non-coding RNAs (lncRNAs) and short non-coding RNAs, which include small interfering RNAs (siRNAs), piwi-interacting RNAs (piRNAs) and microRNAs (miRNAs) [36]. They have epigenetic-related roles that interfere with transcription, translation and mRNA stability [36]. Research focusing on these non-coding RNAs has gained momentum due to their potential roles in regulating heat stress in animals [36]. A study reported numerous microRNAs to regulate the expression of genes associated with cellular responses such as the production of heat shock proteins, immunity, inflammation, cell survival and apoptosis when cattle were exposed to high environmental temperatures [37]. In another study in buffalo heifers, the authors identified the role of miRNAs during heat stress by observing alterations in the regulation of miRNAs, which resulted in changes in the expression of their target genes, such as *HSP60*, *HSP70*, *HSP90*, *HSF1* and *HSPA8*, which were involved in thermoregulation [38]. Recent studies have also demonstrated the role of lncRNAs in regulating thermal stress in animals by influencing genes involved in the cellular response to heat stress and various other biological pathways, aiding in better understanding the underlying molecular mechanisms during heat stress [39,40]. Thus, further studies unravelling the complex epigenetic mechanisms could play a pivotal role in understanding the responses of animals to environmental stressors. This would help in the detection of epigenetic markers, enabling the identification of resilient animals that can endure harsh climatic conditions without compromising their production potential.

## 5. Different Methodologies to Quantify Heat Stress-Associated Epigenetic Changes in Livestock

Advancements in sequencing technologies have made it possible to measure various epigenetic modifications, offering valuable insights into the cellular and molecular mechanisms underlying heat tolerance [28,41]. Methods like DNA methylation profiling, histone modification mapping and non-coding RNA analysis provide a complete understanding of these molecular changes [42]. Therefore, epigenetic data, along with phenotypic traits and gene expression profiles, reveal heat tolerance biomarkers, enhancing livestock management and climate resilience [43].

### 5.1. DNA Methylation Analysis

By mapping DNA methylation changes, biomarkers of heat stress resilience can be identified, which helps in the selection of livestock with an increased capacity to withstand elevated temperatures.

Bisulfite sequencing is considered the “gold standard” for DNA methylation analysis [44], where DNA is treated with sodium bisulfite to convert unmethylated cytosine to uracil, while leaving methylated cytosines unchanged [45]. An alternative, reduced representation bisulfite sequencing (RRBS), focuses on CpG-rich regions, which are the primary sites for methylation [46]. In a comprehensive study of cattle adaptation, an RRBS analysis of blood samples from heat-resilient Nellore and heat-susceptible Angus bulls revealed significant methylation changes in 819 genes during summer heat exposure [6].

On the other hand, whole-genome bisulfite sequencing (WGBS) provides extensive methylation data across the genome, but its use is often limited by the high costs and deep sequencing requirements [47,48]. A recent study on Holstein dairy cows identified 49,861 differentially methylated regions (DMRs) and 7613 differentially methylated genes (DMGs) using WGBS, with significant changes in pathways related to substance transport, oxidative stress and energy homeostasis [42]. For more targeted locus-specific studies, methylation-specific PCR (MSP) and methylation-sensitive high-resolution melting (MS-HRM) offer relatively simple and rapid alternatives [49,50].

Methylated DNA immunoprecipitation (MeDIP) and methyl CpG-binding domain (MBD) capture are two affinity enrichment-based methods that isolate methylated DNA fragments using methyl group-specific antibodies or methyl CpG-binding proteins [51,52]. MeDIP-seq has been used to study how heat stress impacts cattle, showing significant alterations in genes involved in stress management and metabolism [53]. Similarly, MBD capture has been used in bovine mastitis studies, where it has demonstrated effectiveness in isolating heat stress-associated DNA methylation changes [54]. In addition to these methodologies, there are a number of approaches to analyze DNA methylation patterns, most of which have not yet been adopted in studies involving heat-stressed livestock.

### 5.2. Histone Modification Detection Methods

Histone post-translational modifications (PTMs), such as methylation, phosphorylation, acetylation and ubiquitination, play a key role in gene silencing, activation and chromatin remodeling, directly impacting processes like growth, development, disease progression and immune responses [55].

Chromatin immunoprecipitation (ChIP) uses an antibody binding method, where the antibodies bind to specific histone modifications and the immunoprecipitated DNA is then analyzed using PCR, sequencing or mass spectrometry methods to identify the genomic locations of these modifications [56]. There are various ChIP-based approaches, such as ChIP-chip, ChIP-SAGE, ChIP-qPCR and ChIP-seq, which aid in identifying epigenetic modifications [57,58,59].

ChIP-seq has been applied to identify heat-induced histone modifications in poultry in studies such as David et al. [60], which mapped changes in H3K4me3 and H3K27me3 in the hypothalami and muscles of thermally manipulated chicken embryos, revealing genomic regions associated with metabolism, neurodevelopment and stress response.

Furthermore, methodologies like CUT&RUN, cleavage under target and tagmentation (CUT&Tag), mass spectrometry (MS) and the assay for transposase-accessible chromatin with sequencing (ATAC-seq) may also be used to quantify epigenetic modifications [61,62,63,64]. Zheng et al. [32] applied LC-MS/MS to analyze histone modifications in the adrenal tissue of heat-stressed layer chickens, identifying 115 histone markers, with heat-susceptible birds showing elevated H3K27me3 levels.

### 5.3. Non-Coding RNA Profiling

Small RNA sequencing (small RNA-seq) has been particularly valuable in identifying miRNAs and siRNAs involved in heat stress responses across various animal models [65]. Similarly, in dairy cattle, studies by researchers have demonstrated that heat stress alters the miRNA profiles in blood cells and mammary tissues, impacting immune function and cellular processes [66,67]. Additionally, a study identified species-specific miRNA adaptations in milk across different livestock species, providing further evidence of miRNAs’ involvement in stress responses [68].

In addition to small RNA-seq, long non-coding RNAs (lncRNAs) have emerged as crucial regulators of heat stress adaptation in livestock. A study examined lncRNA expression in dairy cattle and identified numerous differentially expressed lncRNAs in tissues like the hypothalamus and pituitary and mammary glands under heat stress. They were found to alter key signaling pathways, including MAPK and mTOR, crucial for stress adaptation and lactation [69]. The chronic effects of heat stress on lncRNA expression in broilers were studied, showing the role of lncRNAs in muscle injury and reduced meat quality under heat stress conditions [70].

**Figure 2:**
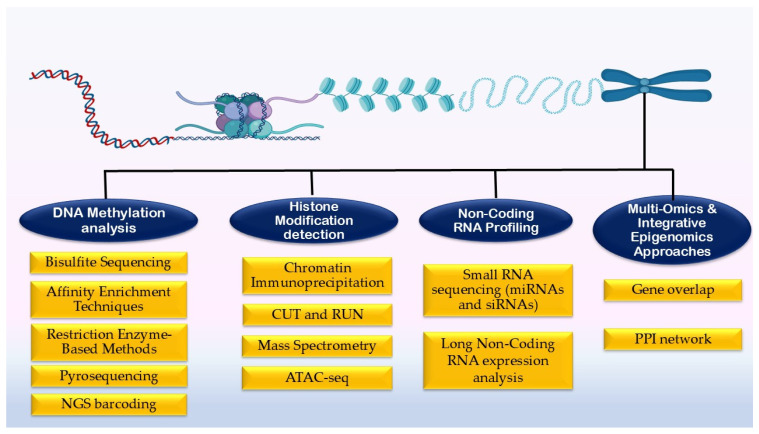
Overview of the different methodologies to quantify epigenetic changes.

Lastly, multi-omics and integrative genomic approaches can play a pivotal role in improving our understanding of gene regulation by combining DNA methylation, histone modifications and transcriptomic data [71]. Mullakkalparambil Velayudhan et al. [72] used a multi-omics approach to evaluate heat stress resilience in two indigenous goat breeds; they employed skin transcriptomics, whole-genome bisulfite sequencing and 16S rRNA metagenomics. The study identified breed-specific thermal adaptation patterns, with Kanni Aadu goats showing 50,560 differentially methylated regions and 7993 differentially expressed genes, compared to 40,648 and 2036 in Kodi Aadu goats. Another study by Reith [73] used whole-genome and targeted bisulfite sequencing together with RNA sequencing to identify heat stress-associated changes in DNA methylation and gene expression in cattle. Such approaches provide valuable insights into the dynamic regulation of genes, offering potential applications in disease prevention, personalized medicine and improving stress resilience in livestock through targeted breeding strategies. Figure 2 provides an overview of the different methodologies used to quantify epigenetic modifications.

## 6. Different Methylome Patterns in Livestock Adaptation to Heat Stress

Animals adapt to their surroundings through complex molecular mechanisms at the morphological, behavioral and cellular levels that help them to cope with the external environment. At the molecular level, genetic variants within regulatory regions modulate gene expression, thereby influencing the adaptation potential [7]. Epigenetic modifications, alongside genetic changes, are key pathways for molecular stress adaptation in animals, allowing gene expression adjustments in response to environmental cues [74]. In particular, DNA methylation may act as a bridge between the environment and the genome, controlling gene expression to generate suitable phenotypic reactions and facilitating long-term adaptation [75].

A study explored the most important pathways in the adaptation of Creole cattle to harsh tropical climates while studying their genomic methylome patterns [76]. The researchers identified approximately 334 differentially methylated regions in the Creole cattle genome, which were connected to candidate genes associated with tropical adaptation mechanisms such as heat resistance, energy management, the immune response, nervous system function and coat and skin attributes [76]. The authors concluded that exposure to significant environmental changes led to alterations in the methylome patterns of Creole cattle, affecting genes tied to key adaptation mechanisms for coping with tropical climates. In a comparative study between heat-tolerant Hariana cattle and heat-susceptible Vrindavani cattle, the authors observed significant variations in their methylome patterns (3845 hypomethylated and 756 hypermethylated CpGs in Hariana cattle) [7]. The authors concluded that the observed epigenetic differences in stress-related genes and microRNAs could play a regulatory role in the long-term heat adaptation and thermotolerance of Hariana cattle [7]. Similarly, in a comparative study between heat-stressed Nellore and Angus bulls, the authors observed breed-specific responses [6]. They observed hypomethylation in the differentially methylated genes of Nellore bulls that were involved in pathways associated with cellular defense and stress responses and suggested this as the reason for the better adaptation of Nellore cattle under heat stress [6].

The long-term adaptation mechanisms during heat stress in small ruminants have also been explored through differences in their methylome patterns. For example, a study established DNA methylation patterns to understand the local adaptation to varying temperatures in Moroccan sheep and goat populations [77]. The authors identified specific differentially methylated regions linked to important attributes such as milk composition, reproduction and thermoregulation. Notably, the authors observed methylation variation in genes such as *AGPAT4* and *SLIT3*, implying epigenetic control over heat-sensitive functions, demonstrating that methylation patterns play a role in local adaptation to temperature variations [77]. In these studies, the identification of distinct methylome patterns in livestock, associated with various adaptation mechanisms and pathways, indicates that DNA methylation contributes to the long-term adaptation of livestock to heat stress.

## 7. Epigenetic Regulation as Response to Heat Stress in Livestock

### 7.1. Large Ruminants

Cattle, a cornerstone of agricultural livelihoods, are vulnerable to heat stress. They exhibit a decline in critical production metrics, including milk yields, reproductive performance and immune resilience. To combat the debilitating effects of climate stress, researchers are actively exploring a broad spectrum of genetic and epigenetic mechanisms to develop innovative, resilience-enhancing solutions.

Research on intrauterine heat stress in Holstein cattle revealed significant epigenetic changes, with approximately 100 genes exhibiting differential methylation patterns in the mammary glands of heat-stressed versus cooled calves. In heat-stressed cattle, the mammary alveolar cells were observed to exhibit a reduced size, potentially attributable to distinct methylation patterns on DNA. Specifically, differentially methylated regions (DMRs) were stated to modulate critical cellular processes, including cell signaling, activation, phosphorylation and protein binding [78]. Differentially methylated genes such as *PRKG1*, *PI4KA*, *PLCB1*, *ASAP1* and *PTK2* were observed to play pivotal roles in orchestrating the complex physiological processes that govern mammary gland development and lactation [78,79]. Notably, reports show that specific genes, including *PRKG1* and *PI4KA*, exhibit hypomethylation, leading to the enhanced regulation of calcium levels and influencing milk synthesis in the mammary gland [78]. In addition, the *AGO2* and *TRIM* genes are upregulated and play a pivotal role in gene transcription and silencing through the regulation of histone deacetylases and chromatin modifiers [78].

The above study further identified DMRs in the livers of heat-stressed bulls, leading to changes in the expression of the *AGER* gene, which codes for the Receptor for Advanced Glycation End-Products (RAGE), a key protein involved in cellular stress responses [78]. Additionally, DMRs were identified in genes such as *ZMAT5*, *ZNF608*, *ZNF395*, *MED1* and *H2AY*, which are implicated in transcriptional regulation and the modulation of innate immune responses [78,80]. Heat stress in pregnant cows was stated to trigger a hypoxic environment, culminating in intrauterine growth restriction, which impairs fetal development and growth [78,81]. This hypoxic condition in the uterus may conceivably alter the methylation status of the *ZNF395* gene in the fetal liver, with potential consequences for the development and function of the innate immune system [78].

Epigenetic modifications indeed converge at a critical juncture, influencing the immunological landscape in heat-stressed animals and potentially impacting their overall resilience to thermal stress [82]. Research on heat-stressed blood mononuclear cells in Holstein cattle revealed the hypomethylation of specific genes, including *IL15*, *BCL2L12*, *HSPB9* and *NDRG1*, suggesting epigenetic reprogramming in response to thermal stress [82]. The identified genes could be potential epigenetic regulators for the heat stress response in cattle as they are involved in varied cellular and immune response pathways, in addition to being molecular chaperones (*HSPB9*).

Recently, researchers found that heat stress triggered epigenetic disruptions in bovine oocytes, characterized by altered histone modifications, DNA methylation patterns and hydroxymethylation levels, ultimately compromising oocytes’ developmental potential [33]. Heat stress has also been shown to modify the function of non-coding RNAs, another vital component of epigenetic regulation. A study in buffalo heifers explored the differential expression patterns of miRNAs during heat stress [38]. The authors observed the significant upregulation of bta-mir-142, bta-mir-1248 and bta-mir-2332 and the downregulation of bta-mir-2478, which were related to genes such as *HSP60*, *HSP70*, *HSF1*, *HSPA8* and *HSP90*. The results suggest that miRNAs could play a potential role in thermotolerance in buffalo heifers [38]. Similarly, Zeng et al. [40] demonstrated that Holstein cattle adapt to heat stress by modulating lactation-related pathways through a network of long non-coding RNAs (lncRNAs). Their study identified specific lncRNAs that regulate genes (*PRLR*, *HSP90B1*, *MAPK8*, *SOC5*) associated with both the cellular heat stress response and physiological processes linked to lactation. Furthermore, Li et al. [83] also investigated the heat stress response in cattle and discovered novel differentially expressed lncRNAs in the mammary glands of heat-stressed cows.

### 7.2. Small Ruminants

Research on heat stress in sheep has revealed a significant increase in N6-methyladenosine (m6A) RNA methylation, which governs the expression of translational proteins, thereby playing a crucial role in the epigenomic response to thermal stress. Being recognized as a key post-transcript landscape supervisor, m6A orchestrates a range of RNA-related events, including alternative splicing, stability, degradation and translation efficiency [84]. In this context, the upregulation of m6A-associated enzymes (YTH domain-containing proteins) in the ovine liver suggests a potential enhancement in RNA methylation, which may serve as a regulatory response to heat stress [85]. However, subsequent findings revealed that heat-stressed Hu sheep exhibited a notable decrease in m6A methylation modification sites per transcript compared to their unstressed counterparts [86].

Emerging evidence suggests that m6A methylation may play a pivotal role in modulating Hu sheep’s response to heat stress, potentially by influencing lipid metabolic genes (*Wnt*, *TGF-β* and *AMPK*) and pathways in the liver and preadipocytes [87]. The author reported a contrasting regulatory role for *METTL3* and *FTO* in the heat stress response, where *METTL3* expression suppresses lipid accumulation via m6A modification, whereas *FTO* promotes lipid accumulation through a similar mechanism. Furthermore, m6A alterations on RNA have been found to exert regulatory control over the expression of heat shock proteins (*HSP60*, *HSP70* and *HSP110*), highlighting a crucial epitranscriptomic mechanism governing thermal stress responses [87,88].

Further studies suggest that epigenetic regulation—specifically DNA methylation—plays a vital role in facilitating the thermal tolerance and environmental adaptability of Hu sheep in regions characterized by extreme heat and humidity [87]. According to a recent study, differentially methylated regions (DMRs) in genes such as *ADCY9*, *PRKACB*, *CREB5* and *TPO* were identified, suggesting a potential epigenetic link to the regulation of thyroid hormone production and thermogenic pathways. Concurrently, methylation events were also observed in the promotor areas of genes including *POMC*, *MC2R*, *ADCY9*, *PRKACB*, *CREB5* and *SP1*, which are pivotal for cortisol biosynthesis [89]. Additionally, a study revealed that heat stress induces epigenetic changes, including allele-dependent DNA methylation, at the *HSP90AA1* promoter, thereby regulating the transcription of this crucial heat shock protein gene [90]. A recent study in Hu sheep revealed an alteration in circRNA expression during heat stress that resulted in lowered reproductive performance [91]. The authors identified 152 differentially expressed circRNAs that were linked with pathways such as apoptosis, mitophagy and the FoxO signaling pathway. The authors suggested that the alterations in the expression patterns of circRNAs during heat stress could be a possible reason for reproductive dysfunction. In another study, heat stress in the livers of Hu sheep led to notable transcriptional changes, including 520 differentially expressed mRNAs and 22 lncRNAs [92]. Among them, *Lnc_001782* was downregulated, along with nearby genes APOA4 and APOA5, suggesting a potential cis-regulatory role in modulating liver function under heat stress [92].

### 7.3. Swine

Thermal stress has deleterious effects on myogenesis and meat quality attributes in swine, compromising the overall value of pork products [93]. Unravelling the epigenetic control mechanisms that regulate heat stress-inducible genes will provide a deeper understanding of the dynamic interplay between environmental stress, epigenetic reprogramming and transcriptional regulation.

A study revealed that heat stress induces widespread epigenetic modifications, resulting in the identification of 1422 genes exhibiting altered DNA methylation patterns in the *longissimus dorsi* muscle [94]. Notably, these DMGs regulate key aspects of skeletal muscle energy homeostasis, encompassing the enzymes 6-phosphofructo-2-kinase/fructose-2,6-biphosphatase (*PFKFB1*), phosphoglycerate kinase (*PGK1*) and pyruvate dehydrogenase kinase isozyme (*PDK3*). The hypomethylation of these genes results in their transcriptional upregulation, which enhances the activity of glycolytic enzymes and restores glucose metabolic homeostasis. Furthermore, heat stress disrupted lipogenic pathways by altering methylation patterns in genes such as carnitine palmitoyltransferase 1B (*CPTIB*), carnitine palmitoyltransferase 1A (*CPTIA*) and leptin receptor (*LEPR*). Heat stress-related epigenetic alterations were also predicted to compromise calcium signaling by affecting the *CLIC2* and *RYR* genes, leading to impaired cellular homeostasis.

Key to porcine skeletal muscle development, the hypomethylation of genes including small muscle protein X-linked (*SMPX*), myosin heavy chain (*MYH11*), collagen type XVI, alpha 1 (*COL16A1*) and collagen type IV alpha 3 (*COL4A3*) was observed under sustained heat stress, highlighting the epigenetic regulation of muscle growth and differentiation. Apart from this, heat stress induced the increased expression of heat shock proteins (*HSP27*, *HSP70*, *HSP90*), *CRYAB* and *DNAJC5*, which played a crucial role in maintaining protein homeostasis by repairing and stabilizing heat-damaged proteins [94].

In addition to the direct impact, maternal heat stress exposure during critical gestational periods has been shown to elicit fetal skeletal muscle gene expression changes, exhibiting a sex-specific pattern and disproportionately affecting female offspring. A comparative analysis of female *longissimus dorsi* muscles showed 282 genes with altered expression profiles in response to heat stress, distinguishing them from control samples. Epigenetic modifications, particularly DNA methylation, likely contributed to the observed transcriptional changes in genes regulating transcriptional silencing (*MTA1*, *NCOR1*, *DMAP1*, *CTBP1*, *EID1*), augmented adipogenic (*PPARGC1B/PGC-1β SREBF1/ADD1*) and fibrogenic pathways (*COL4A2*, *LAMA5*) and impaired angiogenic processes (GO:0016525) [95]. Similarly, Huang et al. [96] investigated the longissimus dorsi muscle in Bama Xiang pigs to analyze the expression of lncRNAs during heat stress. The authors performed RNA sequencing and identified several differentially expressed lncRNAs that were associated with lipid metabolism and muscle development. These differentially expressed lncRNAs could have been the reason for the decrease in meat quality from heat-stressed pigs, which highlights the role of epigenetic regulation as a response to heat stress [96].

Furthermore, a study reported that the exposure of porcine oocytes to heat stress caused changes in the methylation status of adenosine residues (m6A), highlighting a potential epigenetic response to thermal stress [97]. This process is mediated by the upregulation of *YTHDF2* and *METTL3*, key regulators of m6A modification, alongside the concurrent downregulation of FTO, a demethylase enzyme.

Ni et al. [98] performed RNA sequencing in lactating sows to investigate the effects of heat stress on lactation performance and the hypothalamic–pituitary–mammary (HPM) axis. The study identified the significant differential expression of 26, 126 and 169 lncRNAs in hypothalamus, pituitary and mammary gland tissue, respectively, along with notable changes in mRNA expression profiles. A co-expression network analysis highlighted the lncRNAs *MSTRG.17186* and *MSTRG.5366*, as well as the mRNAs *CITED4* and *ROBO1*, as potential regulators of lactation under heat stress [98]. Furthermore, Yu et al. [99] investigated the role of long non-coding RNAs (lncRNAs) in heat stress-induced intestinal inflammation in crossbred pigs exposed to constant heat stress for 1, 7 and 14 days. Their analysis revealed 87, 79 and 55 differentially expressed lncRNAs in the colon at each respective time point, alongside several differentially expressed genes. Through a lncRNA–mRNA interaction network analysis, the study identified several novel lncRNAs, including *MSTRG.13202.5*, *MSTRG.28207.43*, *MSTRG.30039.11*, *MSTRG.34871.3*, *MSTRG.47709.5*, *MSTRG.50167.1* and *MSTRG.8273.18*, that were significantly upregulated in heat-stressed pigs. These lncRNAs were predicted to regulate key target genes, such as *CCN1*, *CLDN1*, *KRT85*, *S100A12*, *NR4A1* and *TM7SF2*, which are implicated in intestinal inflammatory responses triggered by heat stress [99].

### 7.4. Chickens

Heat stress in young chicks alters the body temperature and epigenetically modulates *CRH* expression in the hypothalamic paraventricular nucleus, a key stress response regulator. A report revealed distinct epigenetic patterns in resilient and vulnerable chicks, characterized by reduced CpG methylation and increased hydroxymethylation in CRH introns among resilient individuals [100]. In contrast, vulnerable chicks exhibited elevated CpG methylation and decreased hydroxymethylation. Furthermore, severe heat stress induces post-translational histone modifications, including elevated H3K27ac acetylation, which in turn upregulates *CRH* gene expression by enhancing chromatin accessibility [100].

Temperature modulation during avian prenatal development can reshape the thermoregulatory response, altering the balance between heat production and dissipation and setting a new baseline for thermal homeostasis. In this regard, a study found that the thermal manipulation of naked-neck chicken embryos resulted in epigenetic modifications—specifically, the hypermethylation of the *HSP90α*, *HSP90β* and *HSP70* genes in the brain—which influenced their transcriptional activity at 42 days of age, as compared to control birds [101].

Additional reports revealed that maternal heat exposure was found to alter DNA methylation patterns in chicken embryos, with 289 differentially methylated sites identified, including increased promoter hypermethylation, potentially regulating heat-responsive gene expression [102]. Similarly, a study identified distinct chromatin modification patterns in the hypothalamus, characterized by 785 differential H3K4me3 and 148 differential H3K27me3 peaks, which converged on genes orchestrating neurodevelopment, metabolism and transcriptional regulation [60].

Heat conditioning in chicks also altered histone modifications, increasing H3K9 acetylation and decreasing H3K9 demethylation, which facilitated *Eif2b5* promoter tagging and enhanced *Eif2b5* mRNA expression in the preoptic anterior hypothalamus [103]. Later, the DNA methylation patterns in the farthest reaches of the *HSP70* promoter region of the anterior hypothalamus were investigated, shedding light on the epigenetic regulation of the heat shock response [104]. Furthermore, diminished POU2F1 binding and enhanced NURD complex activity converged to histone H3 acetylation at the *HSP70* promoter, distinguishing severe from moderate heat stress responses [104].

Early-life heat acclimation in chickens, specifically on days 3 or 5, enhanced thermal tolerance by day 10 through epigenetic reprogramming, including DNA methylation at key genes like *BDNF* and *DNMT3A* in the hypothalamus [105]. According to a study, changes in H3K27me3 levels in the adrenal gland were linked to its endocrine function, suggesting a potential role in enhancing heat tolerance in the L2 strain of chickens [32].

Over the years, research has been aimed at *in ovo* heat conditioning to distinguish between maternal influences and epigenetic inheritance. A study demonstrated the feasibility of transgenerational inheritance by showing that *in ovo* embryonic heat conditioning (EHC) enhanced thermal resilience and immune function in subsequent generations [106]. A genome-wide analysis revealed widespread differential methylation in the anterior preoptic hypothalamus, with enrichment in enhancers, CCCTC-Binding Factor (CTCF) sites and genes linked to heat stress (*HSP25*) and the immune response (*SOC3S*), underscoring the intricate relationship between epigenetics and transgenerational inheritance.

Furthermore, Liu et al. [70] have demonstrated alterations in the response of another key epigenetic regulator: long non-coding RNA expression in broiler chickens subjected to chronic heat stress. The authors identified lncRNAs that were involved in muscle injury in heat-stressed chickens. Using transcriptome analysis, the authors detected 68 significant lncRNAs that upregulated apoptosis and fibrosis-related pathways, leading to cellular apoptosis and muscle injury. This resulted in a reduction in the breast muscle yield and meat quality in broilers during heat stress.

Table 1 provides an overview of the varied epigenetic alterations reported in heat-stressed livestock.

## 8. Conclusions

Climate change has significant detrimental impacts on livestock production. Animals adopt various coping mechanisms to withstand harsh climatic conditions, especially through epigenetic modifications like DNA methylation, histone modifications, chromatin remodeling and non-coding RNAs. These modifications play a pivotal role in regulating gene expression in response to different environmental stimuli, which helps in the long-term adaptation of livestock to changing climatic conditions. The tremendous advancements in recent years have enabled the study of these epigenetic modifications, unraveling their underlying complexities. These studies have aided in better understanding the complex molecular mechanisms and pathways behind adaptation traits and have helped in identifying potential epigenetic regulators (*PRKG1*, *PI4KA*, *AGER*, *IL15*, *BCL2L12*, *HSPB9*, *NDRG1*, *DERL3*, *GCLC*, *PPP1R15A*) for the heat stress response in animals. These identified epigenetic regulators can be incorporated into breeding programs as biomarkers through marker-assisted selection (MAS) to identify and develop climate-resilient animals, thereby improving livestock’s productivity and health amid climatic shifts. Furthermore, through nutritional interventions, especially supplementing with diets rich in methyl donors like folate and choline, can positively influence gene expression linked to stress tolerance. Optimal environmental conditioning in the early stages of life, such as maternal care and low-level stress exposure, will epigenetically prepare animals for enhanced stress responses later in life. In addition, transgenerational epigenetic inheritance enables stress adaptations acquired by parents, particularly through optimized nutrition or modulated stress exposure, to be transmitted across generations, increasing population-level resilience. Such integrated efforts will help to improve the resilience of livestock to climatic stressors and in ensuring food security to meet the demands of the growing human population.

## 9. Future Perspectives

**Figure 3:**
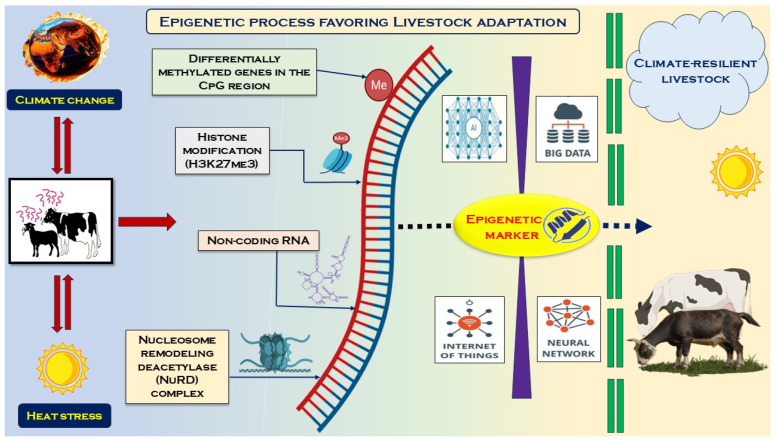
Epigenetic modulation as an approach to the identification of climate-resilient livestock for production.

Epigenetics involves functionally relevant modifications at the genomic level that do not cause changes in nucleotide sequences. It is also thought arise from host–environment interactions, wherein environmental factors influence epigenetic modulation. Therefore, assessing the role of epigenetic modulation in heat-stressed animals could reveal the hidden intricacies of the cellular and molecular responses to heat stress in animals. This field of science, however, is less explored. While studies associating epigenetics with livestock responses to heat stress and adaptation provide intriguing results, the expansion of such research should be emphasized. There are reports indicating a possible association with stress effects on the gut microbiome, consisting of metabolites that can act as epigenetic regulators. Studies in this respect are in their infancy but carry high potential. Likewise, with the rapid advancements in technology, linking artificial intelligence and machine learning with basic research and its findings (for instance, biomarkers for epigenetic regulation in heat-resilient animals) could advance the field. Such integrative approaches, while complex, can yield more accurate results. Given the difficulties associated with genetic improvement in livestock breeding, future programs must focus on multi-disciplinary and multi-institutional approaches. Given the potential of epigenetic markers for resilience to heat stress in livestock, the incorporation of epigenetics in heat stress studies in livestock may be considered to target the selection of climate-resilient livestock (Figure 3).

## Figures and Tables

**Table 1:** Overview of epigenetic modifications in heat-stressed livestock and potential biomarkers.

Species	Breed	Organ/Tissue (Sample Size)	Epigenetic Modifications	Genes/Alleles	Pathways	Reference
Cattle	Holstein	Mammary gland (7)	DNA methylation (hypomethylation)	*PRKG1*, *PI4KA*, *AGO2*, *TRIM*	Regulation of calcium levels, influencing milk synthesis, gene transcription and silencing	[1]
		Liver (10)	DNA methylation	*AGER*, *ZMAT5*, *ZNF608*, *ZNF395*, *MED1*, *H2AY*	Mediates cellular stress response, transcriptional regulation and innate immune defense	
	Nellore	Peripheral blood mononuclear cells (25)	DNA methylation (hypomethylation)	*ELOVL5, FADS1*, *MAP3K1*, *NTN1*, *RASA3*, *PRDX*, *PDE5A*	Regulating immune and inflammatory pathways	[6]
	Angus	Peripheral blood mononuclear cells (25)	DNA methylation (hypermethylation)	*ATG16L2*, *CACNA1C*, *GADD45A*	Modulating cell autophagy, calcium signaling and genomic integrity	
	Holstein	Peripheral blood mononuclear cells (12)	DNA methylation (hypomethylation)	*IL15*, *BCL2L12*, *HSPB9*, *NDRG1*	Vital role in the immunological landscape and chaperone-like activity	[82]
			DNA methylation (hypermethylation)	*APC2*, *BNIP3*	Regulating tumor metastasis and autophagy	
	Hariana and Vrindavani	Peripheral blood mononuclear cells (6)	DNA methylation	*DERL3*, *GCLC* and *PPP1R15A*	Signature genes for adaptative traits and stress response	[7]
				bta-miR-107, bta-miR-1284, bta-miR-2326, bmiR-2396, bta-miR-2441, bta-miR-342, bta-miR-411c-5p, bta-miR-6121-3p and bta-miR-885	Modulation of stress responses through methylation of microRNA genes	
	Holstein	Hypothalamus, pituitary and mammary gland tissue (6)	Non-coding RNA	*MSTRG.6147.5*, *MSTRG.8643.1*, *MSTRG.6147.5*, *MSTRG.8643.1*, *MSTRG.8646.1*, *MSTRG.12225.1*, *MSTRG.16080.1*, *MSTRG.16082.2*, *MSTRG.16082.1*, *MSTRG.16081.1*	Cellular response to heat stress and physiological processes related to lactation	[40]
Buffalo	Murrah Heifers	PBMCs (12)	Non-coding RNA	bta-mir-142, bta-mir-1248, bta-mir-2332 and bta-mir-2478	Regulating thermotolerance	[38]
Goat	-	Ear biopsy (21)	DNA methylation	*AGPAT4*	Lipid synthesis in milk production	[77]
Sheep	-	Ear biopsy (22)	DNA methylation	*SLIT3*	Influences muscle development	[77]
	-	Lymphocytes (16)	DNA methylation	*HSP90AA1* promotor	Regulating the transcription of heat shock protein genes	[90]
	Hu	Blood (20)	DNA methylation (hypermethylation)	*ADCY9*, *PRKACB*, *CREB5*, *TPO*	Pathways repressing thyroid hormone secretion and thermogenesis	[89]
				*POMC*, *MC2R*, *ADCY9*, *PRKACB*, *CREB5* and *SP1*	Modulating ACTH–cortisol signaling loop	
	Hu	Hepatocytes and preadipocytes (3)	m6A RNA methylation	*Wnt*, *TGF-β*, *AMPK*, *HSP60*, *HSP70*, *HSP110*	Influences lipid deposition and exerts control on heat shock proteins	[87]
	Hu	Ovaries (6)	Non-coding RNA	*152 differentially expressed circRNAs*	Apoptosis, mitophagy and FoxO signaling pathway	[91]
	Hu	Liver	Non-coding RNA	*Lnc_001782*	Regulates liver function	[92]
Swine	DLY Pigs (crossbreeds between Landrace × Yorkshire sows and Duroc boars)	*Longissimus dorsi* (16)	DNA methylation	*PFKFB1*, *PGK1*, *PDK3*, *CPTIB*, *CPTIA*, *LEPR*, *CLIC2*, *RYR*, *SMPX*, *MYH11*, *COL16A1*, *COL4A3, HSP27*, *HSP70*, *HSP90*, *CRYAB*, *DNAJC5*	Alterations in DNA methylation patterns within genes involved in energy homeostasis, lipid metabolism, cellular protection mechanisms and calcium signaling pathways	[94]
	Large White × Landrace	Fetal *longissimus dorsi* (LD) muscle (8)	DNA methylation	*MTA1*, *NCOR1*, *DMAP1*, *CTBP1*, *EID1*, *PPARGC1B/PGC-1β*, *SREBF1/ADD1*, *COL4A2*, *LAMA5*	Regulatory pathways involving transcriptional silencing, adipogenesis, fibrogenesis and angiogenesis along with sex-specific differences in gene expression	[95]
	-	Oocytes	m6A RNA methylation	-	Mediated by regulators of m6A modification, especially YTHDF2, METTL3 and FTO	[97]
	Crossbred pigs	Colon (6)	Non-coding RNA	MSTRG.13202.5, MSTRG.28207.43, MSTRG.30039.11, MSTRG.34871.3, MSTRG.47709.5, MSTRG.50167.1 and MSTRG.8273.18	Regulation of intestinal inflammation	[99]
	-	Hypothalamus, pituitary and mammary gland (6)	Non-coding RNA	MSTRG.17186, MSTRG.5366	Regulation of lactational performance	[98]
	Bama Xiang pigs	Longissimus dorsi muscles (10)	Non-coding RNA	365 lncRNAs were identified	Muscle development and lipid metabolism	[96]
Chicken	Cobb	Anterior hypothalamus	DNA methylation	*BDNF* and *DNMT3A*	Dynamic DNA methylation changes in BDNF gene promoter occurred during thermal adaptation, suggesting epigenetic regulation of neurotrophic factors	[105]
		Preoptic anterior hypothalamus	Histone modifications (H3K9 acetylation and H3K9 dimethylation)	*Eif2b5*	Key regulator of global protein synthesis during thermal adaptation, highlighting a dynamic epigenetic mechanism	[103]
		Anterior hypothalamus	DNA methylation, chromatin modifiers (NURD remodeling complex)	*HSP70*	An epigenetic marker of the heat stress response, revealing a molecular basis for thermotolerance variability	[104]
	Naked chicken	Brain tissue (6)	DNA methylation (hypermethylation)	*HSP90α*, *HSP90β* and *HSP70*	Epigenetic regulation of promotor genes for stress response	[101]
		Paraventricular nucleus	DNA methylation, DNA hydroxymethylation, histone modification (H3K27ac)	*CRH* introns	A dual epigenetic mechanism, comprising histone modifications and DNA methylation, within the *CRH* gene-regulatory region, influences stress resilience or vulnerability later in life	[100]
	Broiler	Hypothalamus (54)	Histone modification (H3K4me3 and H3K27me3)	-	Molecular memory of environmental exposure, influencing thermal adaptation in chickens during later life	[60]
	Layer type (L2 strain)	Adrenal gland (192)	Histone modification (H3K27me3)	-	Modulation of adrenal H3K27me3 correlates with adrenal function and may play a crucial role in regulating thermotolerance in chickens	[32]
	Cobb strain broiler	Anterior preoptic hypothalamus (80)	DNA methylation	*HSP25*, *SOCS3*	Conferring transgenerational mechanisms and enhancing both thermal tolerance and immune resilience in offspring	[106]
	Broiler	Muscles (12)	Non-coding RNA	68 lncRNAs	Apoptosis and fibrosis-related pathways	[70]

## Data Availability

Not applicable.
